# Revealing hidden information in osteoblast’s mechanotransduction through analysis of time patterns of critical events

**DOI:** 10.1186/s12859-020-3394-0

**Published:** 2020-03-18

**Authors:** Gianluca Ascolani, Timothy M. Skerry, Damien Lacroix, Enrico Dall’Ara, Aban Shuaib

**Affiliations:** 1 Department of Oncology and Metabolism, University of Sheffield, Sheffield, UK; 2 Insigneo Institute of In Silico Medicine, University of Sheffield, Sheffield, UK; 3 Department of Mechanical Engineering, University of Sheffield, Sheffield, UK

**Keywords:** Osteoblast mechanotransduction, Molecular network, Fluctuations, Subordination theory, Directing process, Waiting time distribution

## Abstract

**Background:**

Mechanotransduction in bone cells plays a pivotal role in osteoblast differentiation and bone remodelling. Mechanotransduction provides the link between modulation of the extracellular matrix by mechanical load and intracellular activity. By controlling the balance between the intracellular and extracellular domains, mechanotransduction determines the optimum functionality of skeletal dynamics. Failure of this relationship was suggested to contribute to bone-related diseases such as osteoporosis.

**Results:**

A hybrid mechanical and agent-based model (Mech-ABM), simulating mechanotransduction in a single osteoblast under external mechanical perturbations, was utilised to simulate and examine modulation of the activation dynamics of molecules within mechanotransduction on the cellular response to mechanical stimulation. The number of molecules and their fluctuations have been analysed in terms of recurrences of critical events. A numerical approach has been developed to invert subordination processes and to extract the direction processes from the molecular signals in order to derive the distribution of recurring events. These predict that there are large fluctuations enclosing information hidden in the noise which is beyond the dynamic variations of molecular baselines. Moreover, studying the system under different mechanical load regimes and altered dynamics of feedback loops, illustrate that the waiting time distributions of each molecule are a signature of the system’s state.

**Conclusions:**

The behaviours of the molecular waiting times change with the changing of mechanical load regimes and altered dynamics of feedback loops, presenting the same variation of patterns for similar interacting molecules and identifying specific alterations for key molecules in mechanotransduction. This methodology could be used to provide a new tool to identify potent molecular candidates to modulate mechanotransduction, hence accelerate drug discovery towards therapeutic targets for bone mass upregulation.

## Background

Osteoporosis is a skeletal disease, characterised by increased probability of bone fractures, that costs the NHS 1.8 billion per year, with a projected increase in cost rising to over £2.2 billion per year in 2025 [1]. The disease is a product of perturbed bone remodelling (BR) process leading to aberrant bone architecture, suboptimal extracellular matrix (ECM) properties and reduction in bone mass [2, 3]. Consequently reduction in bone strength is observed leading to an increased fracture risk in connection with falls in domestic circumstances. BR is driven by the activity of osteocytes (OCy), osteoblasts (OB) and osteoclasts (OC) in response to mechanical and biochemical stimulation. OCs are involved in bone resorption, while OBs drive bone formation and were shown to sense mechanical stimulation [4]. Nonetheless it is widely believed that OCys are the primary mechanosensors in bone, integrating bio-mechanical signals in their microenvironment to orchestrate bone resorption and formation within the BR process. This requires transduction of extracellular signals to coordinated cellular responses; ultimately modulating the ECM’s material properties. Though the mechanisms governing BR has been under examination for at least 50 years, however, the intricate interplay between its components at the molecular and cellular level is not well understood [2, 3, 5–7]. Hence, improved understanding of the mechanisms to transduce mechanical stimuli (mechanotransduction) into cellular activities is fundamental to better comprehend the development of osteoporosis and the effect of related treatments [8–10].

Deformation or strain caused by mechanical loading propagates through bone structure, is detected by OBs, and primarily OCy which are embedded within the bone, via mechanoreceptors such as integrins [11–14]. After mechanosensation the mechanical signal is transformed into cellular responses via the process of mechanotransduction. Integrins are pivotal for OB activation and expression of osteogenic ECM proteins, driving osteogenic differentiation [2, 3, 15, 16]. Stretching and compression of non-homogeneous tissue locally depends on the mechanical properties of the ECM milieu, which arise due to ECM’s protein composition, and consequently affects OCys and their mechanotransduction. Therefore, it is believed that mechanotransduction of the different populations of OBs and OCys drives the orchestration between BR and the process of bone adaptation over time [17].

Mechanotransduction has been investigated with experimental [8, 18, 19] and computational [20–24] approaches; but many aspects of this interaction in bone cells have not yet been explored. This is due to the experimental limitation in modelling physiological molecular events in live cells and the lack of accurate experimental techniques to validate the computational models that could predict such events [25]. Most in vitro data are obtained using stretched cells in monolayer cultures, these may have different ECM relationships to living bone, while measurements based on traction force microscopy are affected by low spatial resolution and constraints in the orientation of applied forces [26]. Conversely, in silico molecular kinetics models using molecular dynamics (MD), ordinary differential equations (ODEs) and partial differential equations (PDEs) are limited to a simulated time which is in the order of 100 ns [27, 28]. Moreover, continuous approaches lack the possibility to consider discrete and highly heterogeneous systems that can form local recurrent structures among the molecules [29–31].

To address these issues, in this study a hybrid mechanical-agent based model (Mech-ABM) was used [32] and a numerical technique for analysis of intracellular events was developed to examine combined bio-mechanical stimulations. The advantage of using an ABM consists in generating a complex dynamics based on simple rules for the physical interactions that would not be easily obtainable in a continuous representation of the system and would be experimentally difficult to control [33, 34]. ABMs are suitable to mimic 3D cellular systems where the dynamics of the signalling and the occurrence of intercellular events can be tracked at the molecular scale. Furthermore, ABMs of various interacting molecules are capable of reproducing local heterogeneity with respect to the number of molecular species, although at larger spatial scale the system appears homogeneous. In our model the heterogeneity of molecular populations responding to directional and time modulated stimuli can be considered [35, 36].

The possibility of having a specific subpopulation of such molecules equal to zero, which is not a global behaviour of the system, cannot be reproduced well with molecular differential equation or with gross grained compartmentalization of the space. Indeed, the scarcity of specific populations implies that specific reactions are excluded. The interaction range gives the unit of measure that defines what is local in such models. The challenge with ABMs is that the smaller the range, the larger the computational cost to identify which molecules interact. Stochastic activation of agents given by their internal clocks add another source of indetermination to which reactions are available for an agent at a specific time and place. This is highlighted with the utilisation of Mech-ABM to examine the link between integrin mechanical properties, mechanotransduction and cell-ECM interaction. The model forecasted that heterogeneity of integrin population is influential in modulating cellular response to mechanical excitation and the emergence of molecular mechanical memory [32]. The aim of this study was to use the Mech-ABM of the OB to study the effect of external mechanical stimuli on the local molecular events within this cell.

Specifically, the ABM simulated intracellular mechanotransduction dynamics emerging due to simultaneous modulation of mechanical excitation, the latter was simulated via the mechanical model. The modulations of mechanotransduction pathways due to these mechanical events were examined. This was achieved by analysing the presence of time structure specific to the mechanotransduction network and the analysis of fluctuations produced in the stochastic representation of the non-ergodic ABM system in terms of recurrences. This approach allowed for cutting out fluctuations sequentially at different specific time scales and analyse the results also for a system in an out-of-equilibrium condition perturbed by external mechanical signals sensed by the integrins on the membrane of the OB.

We acknowledge that some assumptions made within the model will impose a level of constraint on how the data is interpreted, for instance that an OB is a spherical cell and that all intracellular molecules involved are homogenously distributed within the cytoplasm and the nucleus. Another limitation is that our data is illustrating the dynamics of a single OB, while bone formation at a spatial location within bone is driven by a collection of OBs. Nonetheless, projecting the complex system of multi variable agents to the single time components does not reduce the system to a trivial process. The spatial component remains essential, and so the model predicts that within the noise of mechanotransduction there is hidden information characteristic to each class of molecule and of the process dynamics.

### Implementation

**Fig. 1:**
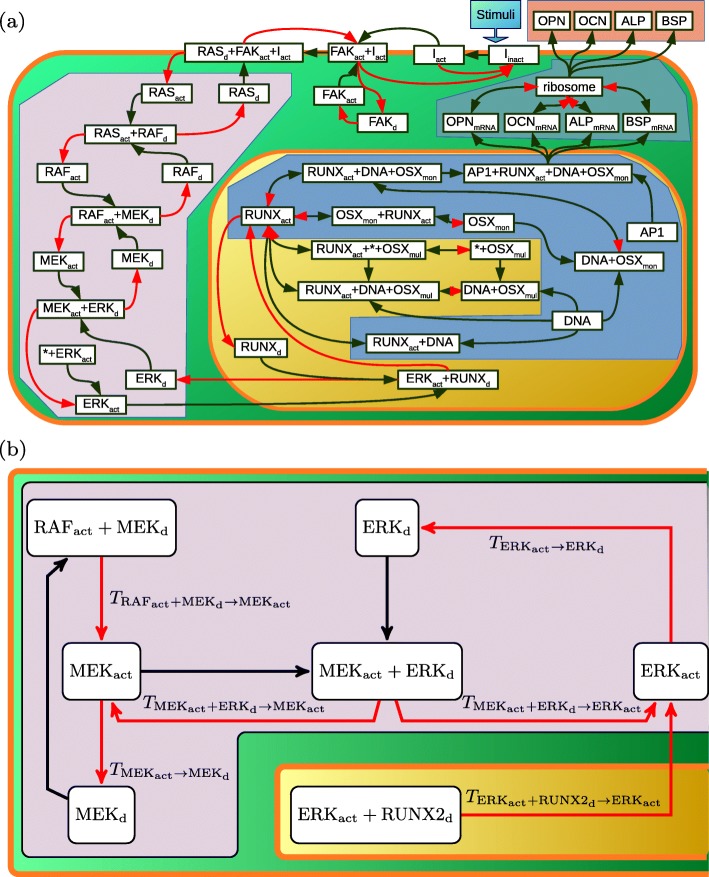
Mechanotransduction molecular network. Schematic representation of all the molecular species simulated in the ABM (white boxes). The arrows show the interactions. Multiple black arrows entering in a white boxes describes mass action reactions, while red arrows coming out (or arrowheads) means dissociation of molecular complexes or energetic relaxation of the molecule. Orange lines separate osteoblast compartments. In the coloured boxes, the network modules are enclosed. (a) Complete diagram with the external stimuli (cyan), the energetic MAPK mod. (pink), the transcription mod. (blue), the translation mod. (green) and the ECM proteins (red). (b) Part of the diagram showing only the simulation dependent delays and the respective molecules. Interactions depicted in black have fixed parameters

We used Mech-ABM to mimic the dynamics in the mechanotransduction pathway of a single OB during the deposition of ECM factors under external mechanical perturbations [32]. Each agent represents a specific molecule, which has a relevant role in the process of ECM formation and is included in the mechanotransduction network (see Fig. 1a). The agents move following a constrained Brownian motion in one of three geometrical compartments - the cell nucleus, the cytoplasm, and the extracellular region surrounding the cell (orange borders in Fig. 1a). When the distance between two agents is shorter than the interaction range (*R*_inter_), a set of rules depending on the internal state of the agents are applied to each agent involved in the interaction. Similarly, single agents can trigger rules due to their internal clock, state or information exchanged. The actions resulting from the application of rules triggered by an agent may involve:

1. The changing of its own state,
2. Self-destruction and degradation,
3. The production of information causing:
  - Other existing agents to change their states,
  - The creation of new agents, or
  - Triggering other actions (e.g. becoming static).

In terms of agents, the reactions between two molecules generating a molecular complex are of two types (Supplementary Fig. S1). The first requires the change of state of one agent; the second involves the change of the state of both agents. Reactions referring to a transfer of a molecular messenger are obtained by the change of the states of the agents involved. Dissociations to two or more molecules of complexes regulated by activation cycles switch (ACS) and molecular persistence in a given state have two ways. One way is the change of state of the original agents leading to appearance of a different states and the full dissociation/break-up of the complex. The other consists of state change of some agents forming the complex, thus only the molecules with changed state dissociate/break-away from the complex and consequently the complex does not fully dissociate.

Among the molecules simulated there are proteins (e.g. integrins), ribosomes, mRNA (messenger RNA) and vesicles. For the sake of simplicity, other elements like amino acids, sophisticated organelles (e.g. Golgi apparatus), molecules and complexes not included in Fig. 1a were excluded. The rules used in the proposed model are based on the affinity of the molecules, presented in said figure, that were derived from chemical reactions [24, 37, 38]. The updating scheme adopted in the model [39] requires that, at each iteration, all the molecules are aggregated and checked for possible rules to be applied. These interactions that drive the model dynamics are either: 1) binary binding of molecules based on one molecule querying all the available molecules in the sphere with radius *R*_*inter*_ and then requesting the interaction with the nearest one; 2) state transition, stabilisation and complex dissociation activated at the expiration of an internal timer set to a random time sampled from a probability function Ψ(t), when the complex changes state and decreased of 1 unit of time at each iteration.

The chemical dissociation of a complex molecule into two components occurring in the absence of other interactions with the surrounding environment due to lack of physical knowledge is described as a Poisson decay process. It follows that the times of occurrence of dissociation events are uniformly distributed. Even though Ψ(t) does not give any prior knowledge of the dissociation events, it is still possible to choose the average and support of the waiting time (WTD) distribution based on experimental evidence [40, 41].

In the simulated Mech-ABM, among various signals, we generated the accumulated number of agents partitioned by their state variables mapping to their equivalent biological attributes, thus represented distinguishable molecular subpopulations (e.g. active/inactive and bound/unbound).

Stochastic processes were integrated within the Mech-ABM, whereby stochastic updates of every agent’s global and local variable over time; specifically the ACS variable, were introduced [39]. Hence, simulated molecules are present in at least two complementary isoforms: active and inactive. In macroscopic steady state conditions, when one isoform produces a smooth signal close to zero axis interrupted by positive spikes, the other generates a negative fluctuation around a positive nominative value. Indeed, the process becomes immediately much more complex when there are not only active and inactive states, but also interactions with other molecular species [5, 7]. Comparing multiple simulations, it can be seen that spikes between different repetitions are not synchronized, and the time intervals between spikes of the same signal are not constant. These fluctuations, which are typical of random processes, can be difficult to associate with specific reactions in presence of a heterogeneous population of agents undergoing different interactions. Nonetheless, formation of patterns and information of the complexity of a non-ergodic system can be extracted.

### Signal transformation

The agents generated by the ABM contain time-dependent information relative to their position, velocity, compartment of origion, molecular activation states, bound state and an internal clock. If each agent has *n* variables, and there are *S* agents, then the state of the system can be represented in a *n × S* dimensional phase space. In order to reduce the dimensionality of the systems phase space, in this study we neglected all the information relative to the position and velocity of the agents and we considered only the molecular activation state, the bound state and, when biologically relevant, the intracellular compartment. These quantities are categorical so we can label them with discrete indices. The signals analysed are the sum of molecules with the same labelling. If each of these quantities are state variables they have values *y*_*i*_(*t*), where *i* is the index for the specific state variable and *y* is the value dependent on the time t. Given the stochastic origin of the simulations, the resulting time series are fluctuating signals, which can be decomposed in two components: the trend, *Y*_*i*_(*t*), and the fluctuations around the trend, (t). We introduced a method to process the signal in order to obtain a positive defined time series of the noisy component by using the time average, a filter that removes the random fluctuations faster than a time lapse ∆_*T*_:

$$ {\overline{y}}_i(t)={\hat{A}}_{\varDelta_T}{y}_i(t)\overset{\varDelta_T\to \infty }{\to }{\overline{Y}}_i(t), $$

where the over bar represents the time averaged signal and the operator is the moving average operator:

$$ {\hat{A}}_T=\frac{1}{\varDelta_T}{\int}_t^{t+{\varDelta}_T}\left(\cdot \right)\; dt\hbox{'}. $$

This approach allowed us to cut out fluctuations sequentially at different specific time scales and analyse the results also for a system in an out-of-equilibrium condition perturbed by external mechanical signals sensed by the integrins on the membrane of the OB.

The periodic stepwise external perturbation changes the equilibrium state according to the frequency. For fast variations compared with the response of the molecules, the system remains out of equilibrium for the whole simulation while the molecules try to reach the equilibrium condition. The average of the signal, being non-constant in time, is one of the reasons why the system is not ergodic. Therefore, the trend from each signal was removed in order to obtain the fluctuations around the trend.

### Subordination theory and detection of events

In this work, we propose a numerical method to disentangle random processes, called subordinated processes [42], into their respective parent process and directing process in order to analyse the latter in terms of patterns of recurrence of events [43]. A subordinate process, mathematically defined in [43–46], can be created by first generating a leading process, which can be can be deterministic or derived from a random distribution X, so that a trajectory x has values *x*(t^*^_i_) at discrete positive times t^*^_i_ with t^*^ − t^*^_i - 1_ = ∆t^*^ *>* 0 *∀ i ∈* N. If we rescale the time such that ∆t^*^ = 1 unit of time, then t^*^_i_ = *i* and the parent process is described by *x*(t^*^_i_) = *x*(*i*) where the steps *x*(t^*^) *− x*(t^*^_i - 1_) are derived from *X*. Then in a similar way a directing process *T* is generated from a random distribution with positive support such that *τ*_*i*_ defines a monotonically increasing function. $t\left({t}_i^{\ast}\right)={\sum}_{j=0}^i{T}_j$ The subordinated function is defined as the trajectory *x*(*t*). Generally t^*^ is called the natural time and t is called the physical time, which is the macroscopic and experimentally observable time. The approach presented necessitated detection of critical events in the system under analysis. The system within the Mech-ABM is inherently fluctuating, leading to variation in the quantity of molecules at a given time. In order to detect the events for a molecule *i*, first, the time series, *y*_*i*_, was filtered with a moving average over a time lapse *τ*_1_ on each state variable, such that *y*_*i*_^*I*^(*t*) = *Â*_*τ*1_ *y*_*i*_(*t*). Then the operator *Â*_*τ*2_ was applied, once more, to the variation of each molecular specie i from its estimated mean ∆*y*_*i*_^*I*^(*t*) = *y*_*i*_^*I*^(*t*) *− y*_*i*_(*t*); the result of which is *y*_*i*_^*II*^(*t*) = *Â*_*τ*2_ ∆*y*_*i*_^*I*^(*t*), where the intervals of time are constrained by *τ*_1_ *> τ*_2_. The first moving average has been used to de-trend the time series from the signal (intended as the component with slower dynamics). The subsequent second moving average has been applied to determine the amplitude *σ*_*i*_(*t*) of the second moment of the non-local noise fluctuations *y*_*i*_^*I*^(*t*) central with respect to *y*_*i*_^*II*^(*t*). The term non-local is intended as non-local in time, and it includes all the times belonging to [*t − τ*_2_*/*2*, t* + *τ*_2_*/*2] for each time t.

**Fig. 2:**
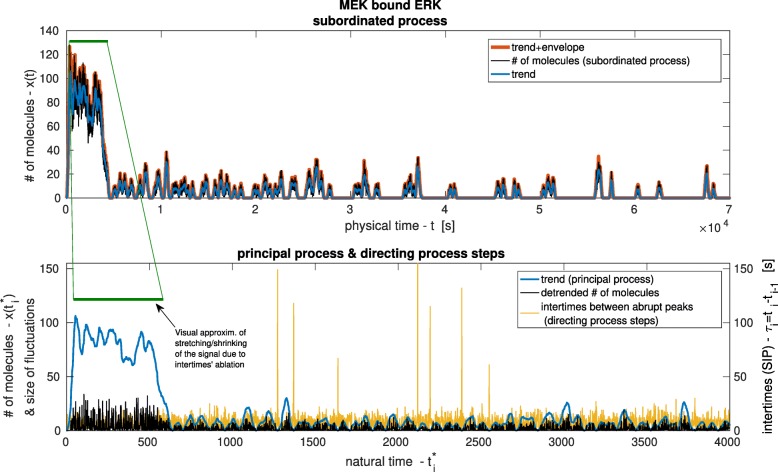
Subordinated process. On the top graph, the amount of molecules for the complex MEK + ERK in function of the time *t* is shown. This curve represents the subordinated signal *x*(*t*). The trend of the signal, derived by time average, and the envelope, derived by Hilbert transform, are used to detect the critical events. In the bottom graph, the signal interim period (SIP) *τ*_*i*_ between two consecutive critical events are shown in function of the physical time *t*_i_^*^ = *i*. The curve *t*(*t*_i_^*^), given by the sum of all the SIP *τ* up to the i^th^ event, is a realization of the directing process. In the same graph, the curve *x*(*t*_i_^*^), given by the sum of all the steps ∆*x* up to the i^th^ event, is a realization of the principal process. For convenience, in the bottom graph, the fluctuations around the trend are shown. In the curve *x*(*t*_i_^*^), regions with high number of events resemble a stretched form of the subordinated signal while regions with few events manifest shrunk portions of the subordinated signal

The time series ∆*y*_*i*_^*I*^(*t*) fluctuates around zero. The Hilbert transform, defined as the integral operator

$$ \hat{H}= PV{\int}_{-\infty}^{\infty}\left(\cdot \right)\frac{d\tau}{\pi \left(t-\tau \right)}, $$

has been used on the de-trended signals to derive the symmetric envelope of the de-trended signal, *y*_env_(*t*) = │*Ĥ y*_*i*_^*I*^(*t*)│, from which it has been subsequently detracted. The result, *y*^*III*^(*t*) = *y*^*I*^(*t*) *− y*_env_(*t*), is a positive defined time series with enhanced prominent peaks and dumped valleys close to the zero axis, Fig. 2. For each component *y*_*i*_^*III*^ of the signal, the peaks are detected as events ℰ, if the signal minus one standard deviation of the fluctuations drops after each peak, *p*_*n*_(*t*), below the corresponding preceding peak value:

$$ \mathrm{\mathcal{E}}=\left\{{p}_n\left({t}_1\right),\exists {t}_2\ |\ {y_i}^{III}\left({t}_2\right)-{\sigma}_i\left({t}_2\right)<{y_i}^{III}\left({t}_1\right)\right\}. $$

The component *y*_*i*_^*III*^ has regions of small values; the beginning and ending of those regions are detected as events if the subtraction of one standard deviation of the fluctuations to the signal drops below or increases above the zero. The WTDs between peaks or the time extent of peaks are considered. To avoid artefacts related to fixed bin size and to be able to compare distributions obtained with different numerical parameters (in general having different binning sizes), we use a Kernel Density Estimator (KDE) defined as:

$$ KDE(t)=\frac{1}{w}{\int}_t^{t+w}K\left(t-\tau \right)\left(\cdot \right)\; d\tau\;\mathrm{such}\ \mathrm{that}\ {\int}_{-\infty}^{\infty }K(t) dt=1,w\kern0.40em \mathrm{is}\ \mathrm{the}\ \mathrm{support}\ \mathrm{of}\ \mathrm{the}\ \mathrm{operator}. $$

### Signal post-processing

The directing process, defined as one component of the subordinated signal and here numerically obtained by detection of critical events, provides information on the recurrences of large fluctuations of the number of molecules rapidly reaching or departing from a given node of the network, Fig. 2. The WTD of the directing process depends on the chosen parameters so it is representative of the specific dynamics of the model. Due to the stochasticity within the model and the applied mechanical stimulation the critical events would be gamma distributed, and characterize by multimodality.

The integral distribution is first derived by weighing each signal interim period (SIP) *τ* between two consecutive events, *y*_1_^*III*^(*t*) and *y*_2_^*III*^(*t* + *τ*), with the amplitude of the fluctuation *y*_1_^*III*^(*t*). The area *τ × y*_1_^*III*^(*t*) is a measure of the minimum cumulated time that molecules in a given state *i*, which have been involved in the same critical event, are going to spend in any state different from *i*. We can see it as the cumulated dispersion time of molecules sharing the same kinematics by departing from the same node of the network.

The non-normalized distribution gave the amount of the total number of molecules with the same averaged dispersion time found inside the entire duration of the sampled signal. All the signals have been de-trended and positively defined. The WTD, *ψ*(*τ*) is the probability density obtained by normalizing the integral distribution to 1.

### Simulations and sensitivity analysis

In order to verify the repeatability of the results, for each set of parameters, we repeated 10 independent simulations. For each simulation, we computed the distributions, and we used the 10 independent results to derive the corresponding punctual confidence interval, (notice that the top and bottom of confidence intervals around the WTDs are not probability functions). Due to the lack of knowledge of the WTDs’ dependency on any parameter, mathematically speaking, the computation of a variation of the distribution ψ is unfeasible by applying an infinitesimal affine transformation depending on a small variation of the parameters [47]. Consequently, the only possible approach consisted in running the stochastic simulations for each set of parameters analysed. This computationally expensive choice was validated by the change in modality of the WTDs shown in the results. Indeed, the model was simulated for sufficiently large variations of the parameters such that visible variations of the WTDs shape were obtained.

Given the large amount of data simulated and results generated, analyses were restricted to integrins, RUNX2, and the RAF-MEK-ERK module (Fig. 1b). These proteins and their interactions are of interest due to their role in propagating mechanical forces from plasma membrane to the nucleus of the OB; their direct involvement in mediating osteogenesis and the regulation of BR [48–50]. The results presented here are focused on the observations associated with the RAF-MEK-ERK module.

The generic ABM platform FLAME (Flexible Large-scale Agent Modelling Environment) was used to simulate intercellular mechanotransduction and mechanical loading [35, 36, 51, 52]. The simulations start with 8890 agents (Table 1) evolving with a unit time step corresponding to 1 s for a period of 9 *·* 10^4^ s (approximatively 24 h). The initial condition for the position and velocity of the agents has been chosen at random from a uniform distribution. The mechanical load used to perturb the system is mimicked by a periodic square wave function defined by its magnitude *M* and oscillation period *P*, while the phase is set to zero, and the minimal magnitude is equal to 100 μPa, see Table 2.

**Table 1:**
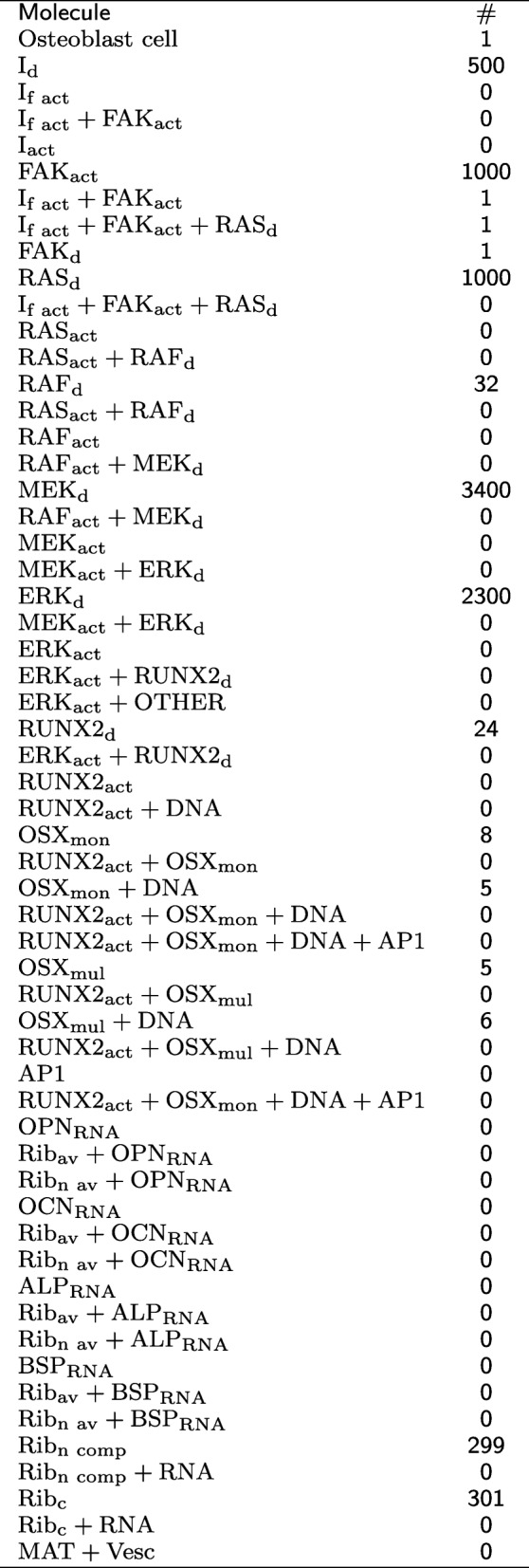
Number of molecules at time t = 0

**Table 2:**
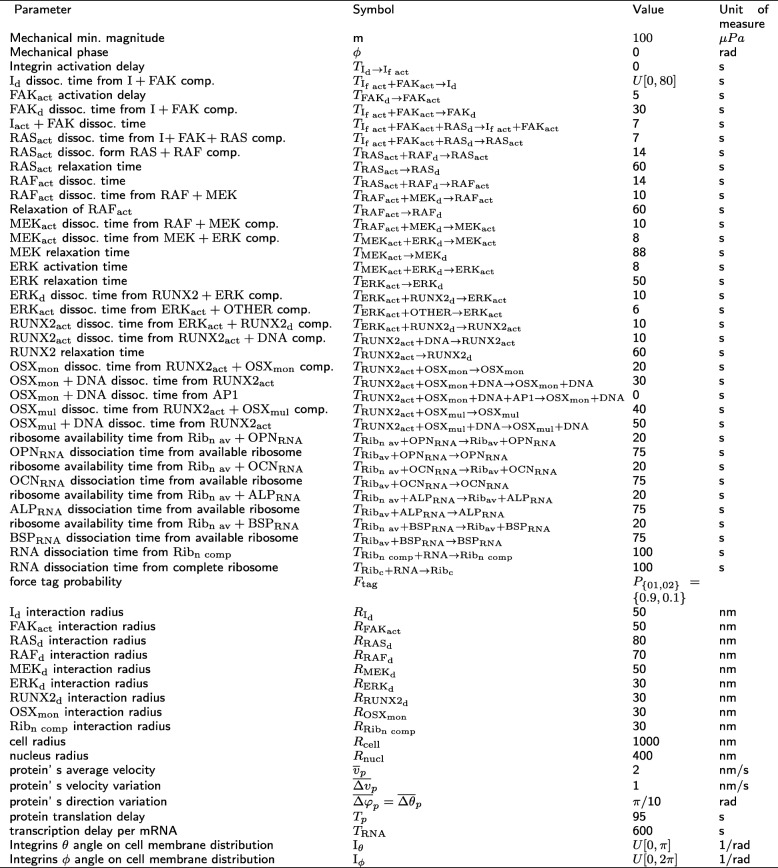
Parameters’ names, symbols and values

The proportions of agents belonging to specific molecular species, at time equal to 0, are constant among all the simulations performed, and their respective values are shown in Table 1. The values of the parameters for the baseline simulations are shown in Table 2.

The simulations were run at the SHeffield Advanced Research Computer cluster (SHARC) on machines with 2 x Intel Xeon E5–2630 CPUs and 64GB of RAM. Each simulation required 8GB of RAM, 96 h of execution time and approximatively 500GB of hard disk space.

The effect on the fluctuations of the molecular populations were investigated by changing the magnitude *M* and oscillation period *P* of the external perturbation and the ACS of the molecular times *T*_*• → •*_ (*• → •* represents the initial and final transition states) for: *T*_RAFact_ _+ MEK__d_ _→_ _MEK__act_, *T*_MEK__act_ _→_ _MEKd_, *T*_MEK__act__ + ERK__d__→_ _ERK__act_, *T*_MEK__act_ _+ ERK__d_ _→_ _MEK__act_, *T*_ERK__act_ _→_ _ERK__d_, *T*_ERK__act_ _+ RUNX2__d_ _→_ _ERK__act_ (Fig. 1(b)).

All combinations for *N*_*M*_ = 2 values of the perturbations magnitude, *N*_*P*_ = 3 values of the perturbations period, and *N*_*T*_ = 5 values for each of the 6 dissociation/ACS times has been analysed, and the explored parameter values were reported in Table 3. Each simulation has been repeated 10 times for a total of 1800 simulations. Analyses and additional results derived by the simulations are stored in Google Drive (https://drive.google.com/drive/folders/1Q0HFuvmv6NTxpBXfKQerR93I-4_t9LAu?usp=sharing).

**Table 3:**
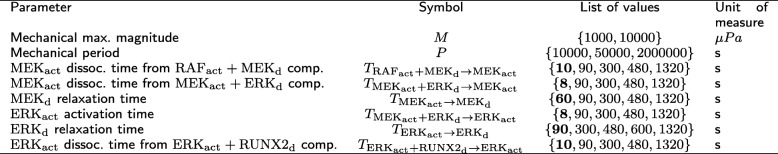
Parameters ranges: Names, symbols, unit of measures and list of values simulated. Bold quantities represent the baseline values. Where no baseline is present, then all possible combinations has been considered. Each set of parameters has been independently repeated 10 times

### Phenomenological model and constraints

In Mech-ABM the number of simulated intercellular molecules is much smaller than in the real biological system, due to the large resources, in terms of memory and computational costs, required for simulating ABMs in magnitude ≥10^9^ of agents. This represents an upper limit in the number of agents which can be simulated. Another limit is given by spurious behaviours introduced by finite size and finite volume effects [53] representing an inferior boundary condition for the number of agents in the model. In our case, we have an average increasing number of agents during the simulation, which limited us in the initial maximum number of molecules. Nevertheless, the model does not explicitly include any finite volume exclusion among molecules. Furthermore, the proportions of some molecules such as integrins in a specific state on the cell membrane are strongly driven by the external perturbation independently from the total number of integrins simulated. This phenomenological aspect overrides many finite size effects. Consequently, we assert that we can rescale the concentrations of the molecules and maintain the same averaged dynamic of the system by rescaling the interaction range *R*_inter_ of the molecules accordingly [36, 53].

## Results

### Waiting time distributions and spectrograms

#### Dissociation times/ activation cycle

**Fig. 3:**
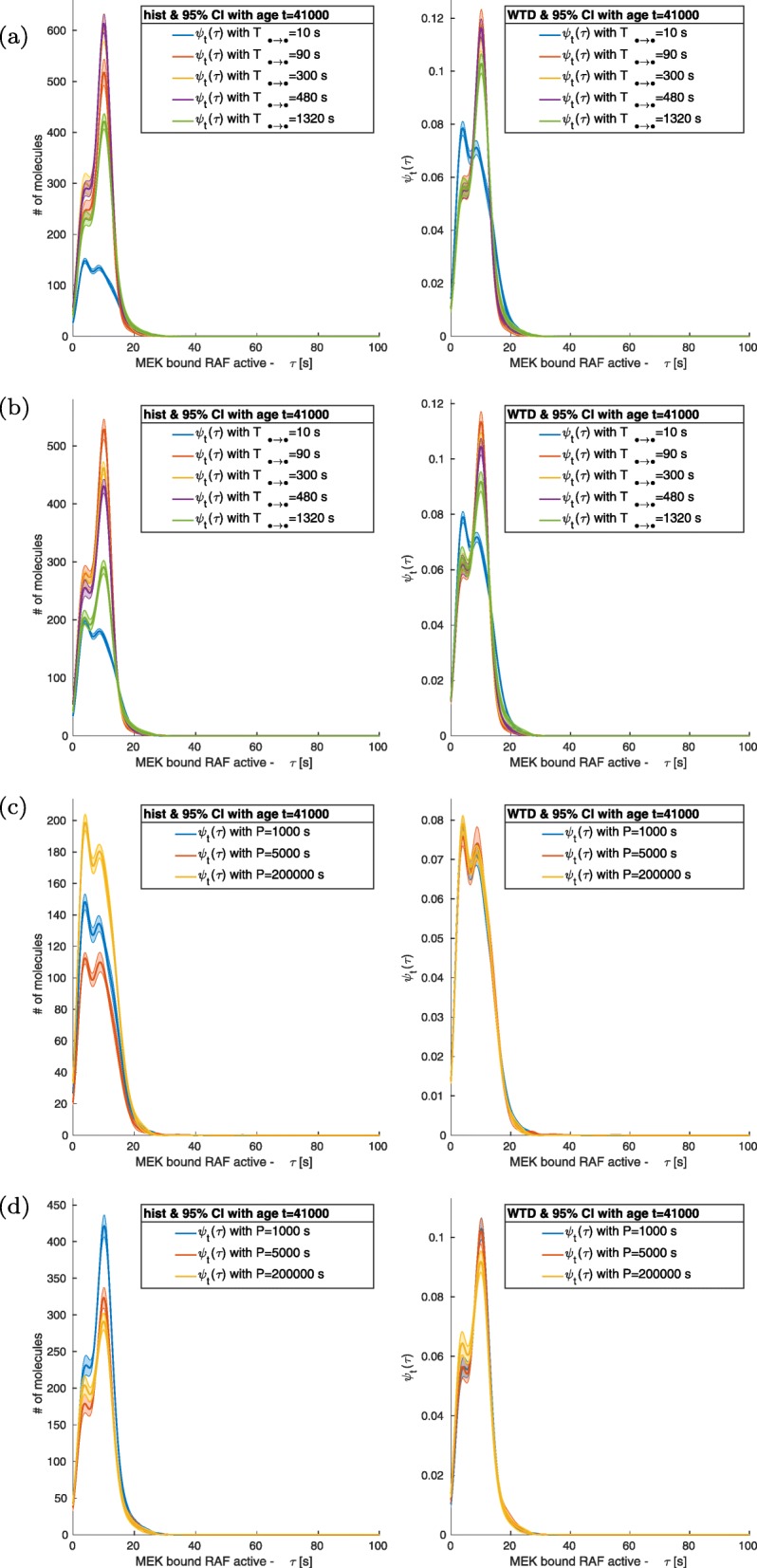
MEK bound RAF. Independently from the frequencies of the tested perturbations, the variation of *T*_RAFact + MEKd → MEKact_ leaves the MAPK sub module of the network unchanged exception for the MEK interacting with RAF which is highly sensitive to the parameter. Indeed, the distribution of recurrence of events for active MEK bounded to RAF is bimodal in *τ* = *{*4*,* 9*} s*. For small value of *T*_RAFact + MEKd → MEKact_ *∼* 10 *s* the distribution is mainly centred around τ = 4 s and it represents the main mode, while for larger value of *T*_RAFact + MEKd → MEKact_ the distribution’s main mode is at *τ* = 9 *s*. **(a)** *T* = 1000 *s*; **(b)** *T* = 200,000 *s*; **(c)** *T*_RAFact + MEKd → MEKact_ = 10 *s*; and **(d)** *T*_RAFact + MEKd → MEKact_ = 1320 *s*

As MEK and RAF interact, Raf-MEK complex forms, MEK is activated by phosphorylation, the Raf-MEK complex dissociates, and consequently activated MEK propagates mechanotransduction. Therefore, activation dynamics of the Raf-MEK complex governed by positive and negative feedback loops dictate the complex dissociation. Hence activation-deactivation cycles are used interchangeably here. This was also observed for other complexes such as MEK-ERK. Figure 3 shows that the fluctuations of the complex RAF-MEK presents two distinct dynamics depending on the dissociation time. For dissociation time *T*_RAFact_ _+ MEK__d_ _→_ _MEK__act_ of approximately 10 s, and varied mechanical oscillatory periods, the WTD is a bimodal distribution with modes around the recurrence times *τ*_1_ = 4 s and *τ*_2_ = 9 s, Fig. 3c. The distribution *ψ*(*τ*_1_) is approximately 15% larger than in *τ*_2_. When *T*_RAFact_ _+ MEKd_ _→_ _MEKact_ is increased from 90 s to 1320 s, the fluctuations dynamics changed; the peak at *τ*_1_ was reduced significantly and the distribution has only one relevant maximum at *τ* = 9 s. At *T*_RAFact_ _+ MEKd_ _→_ _MEKact_ = 1320 s the integrated distributions and the WTD show that a large portion of the Signal Interim Period (SIP) fluctuations are close to the mode and the occurrence of critical events is more regular. For *T*_RAFact_ _+ MEKd_ _→_ _MEKact_ = 10 s, we also see the number of molecules with the same dispersion time is below 200 for any delays and any period (*P*) of the mechanical stimulation, while it is larger than 400 at *τ*_2_ for dissociation times *T*_RAFact_ _+ MEKd_ _→_ _MEKact_ > 10 s.

**Fig. 4:**
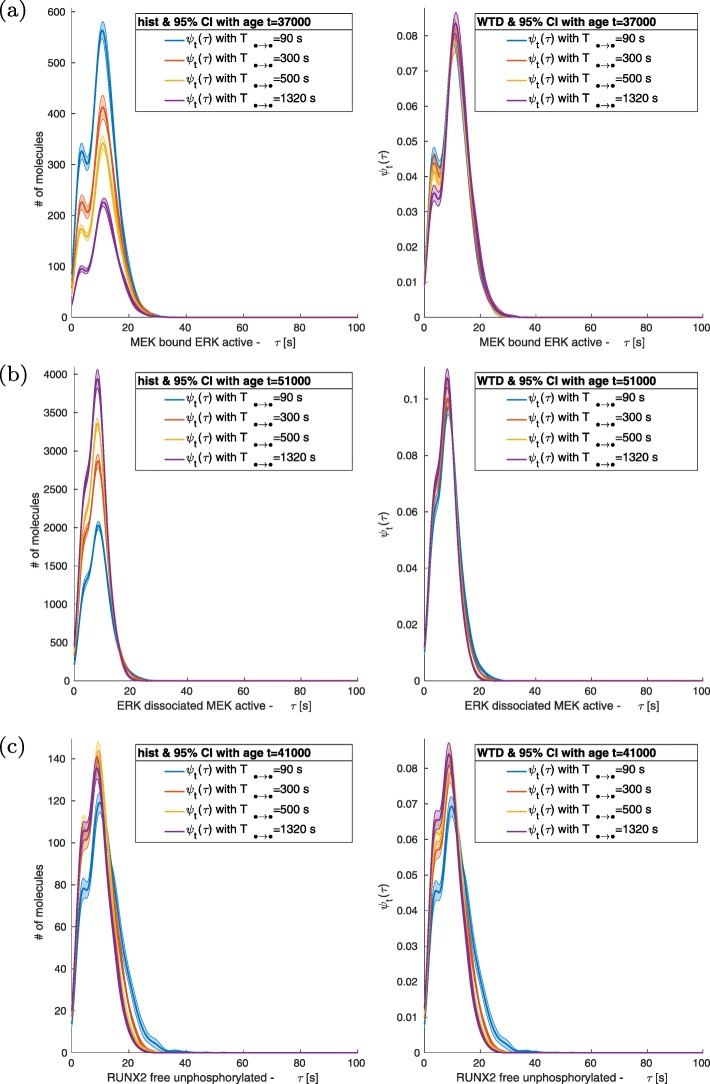
MEK + ERK, ERK and RUNX2. Distributions for different values *T*_ERKact → ERKd_ under a periodic perturbation with *P* = 1000 *s* and *M* = 10,000 μPa. *T*_ERKact → ERKd_ produces larger effects on the next nearest neighbour of the network like unphosphorylated RUNX2 and unphosphorylated ERK. Variations in the distributions and in the non-normalized histogram of MEK bounded to ERK. No effects are visible on the distribution of active ERK dissociated from MEK. Amplitudes or number of fluctuations of ERK dissociated MEK increase with the increase of *T*_ERKact → ERKd_, while they show an inverse relation for MEK bound to ERK decrease

**Fig. 5:**
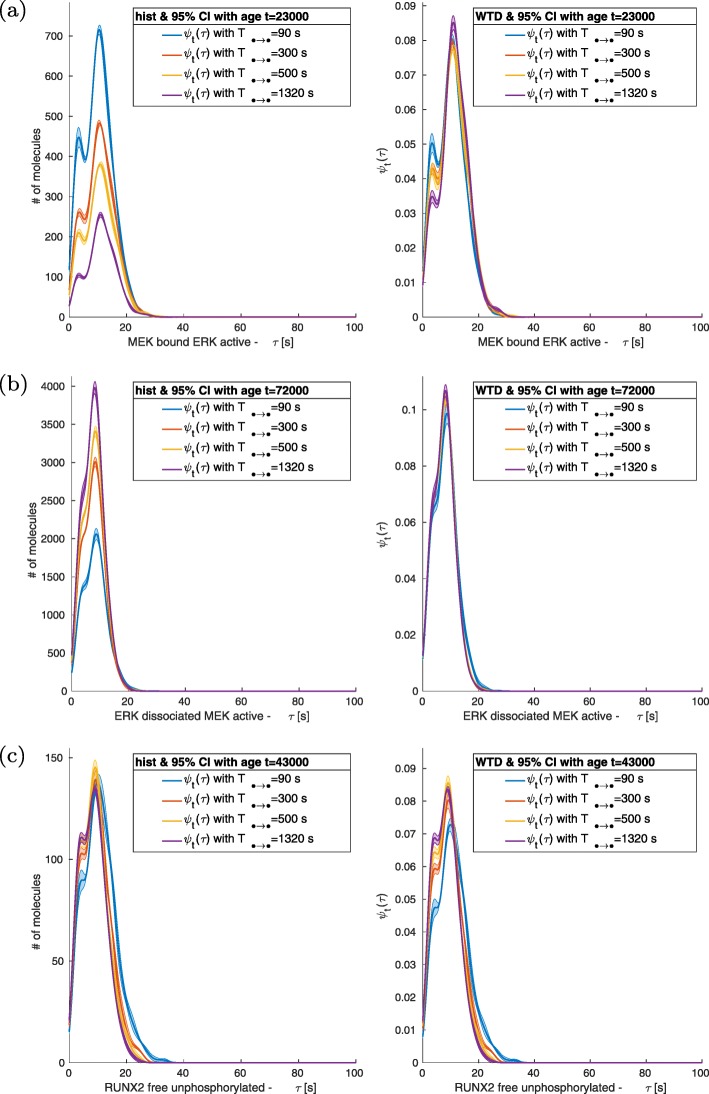
MEK-ERK, ERK and RUNX2. Distributions for different values of *T*_ERKact → ERKd_ under a periodic perturbation with *P* = 200,000 s and M = 10,000 P a. The distributions are similar to those obtained with higher frequency stimulation. The histograms show larger or more frequent fluctuations of unphosphorylated RUNX2 and MEK bound ERK

**Fig. 6:**
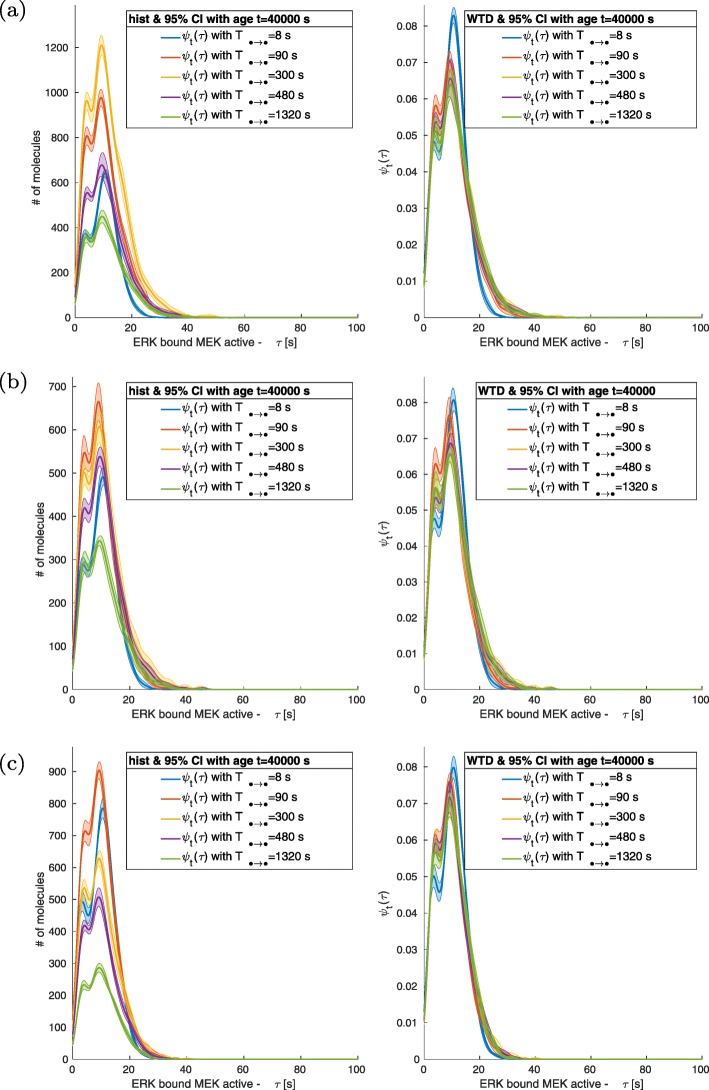
ERK-MEK complex. The distributions of SIP for the ERK + MEK complex WTDs are bimodal. The modes are at T equal to 4 s and 15 s. When *T*_MEKact + ERKd → ERKact_ = 8 s, the mode at T = 15 s shifts to 17 s. The non-normalized histograms on the left column show that the total number of events is at the minimum when *T*_MEKact + ERKd → ERKact_ has the maximum value simulated equal to 1320s. The number of critical events increases when *T*_MEKact + ERKd → ERKact_ decreases and reaches a critical value after which the dissociation time is directly proportional to the number of events

Reducing the values of ACS causes an increase of molecular interactions and a corresponding effect on the number of fluctuations in the signals. Such a phenomenon can be seen in non-normalized histograms of bounded molecular complexes like RAF + MEK, Fig. 4a and Fig. 5a. Instead, free molecules show a direct relation between the size of the fluctuations and the dissociation/deactivation value, Fig. 4b-c and Fig. 5b-c. Nevertheless, we have observed that the tendency of higher amount of events for smaller value of ACS (*T*_*• → •*_ < 1320 s) does not always hold; see Fig. 6 a-c. In such cases, after the initial increase of the number of events, there is saturation. Decreasing ASC (*T*_*• → •*_ < 1320 s) further cause a variation in the dynamics, and the quantity of critical events becomes directly proportional to the value of the relaxation/dissociation time.

#### Mechanical perturbations

The functional form of the external perturbation is a periodic square wave which is completely characterized by the amplitude M and the period P, while the phase is set to zero, and the minimal magnitude is equal to 100 arbitrary unit (au), see Table 2. In the range of values of P investigated, two were much shorter than the total duration of each epoch, and in one case the period was set so that the stimulation applied was constant for the entire simulation. The external force activated mechanotransduction cascade and the dynamic of the system which, otherwise, would remain trapped in its initial condition. For all the analysed epochs and the simulated periods, the results indicated that the perturbation function as a switch. When the perturbation goes to its minimum, integrin activation approaches zero, and eventually other molecules in the pathway follow this trend. For all the molecules which are downstream in comparison to the integrins, the larger their network distance is, the larger their delays are in following the dynamics of the external perturbation (Fig. 1a).

The passage of the mechanical loads from high to low and vice versa is of the order of 1 time unit (1 s), and the duration P of each regime is at least 1000 s long (see Table 2 and Table 3). Indeed, all the WTDs analysed have a support shorter than all P considered, and in Figs. 3, 4, 5 and 6, the recurrence of events does not require a time larger than 100 s.

The paucity and sparsity of the events characterized by the high/low transitions of the external perturbation do have a minor effect on the SIP *τ* between molecular events, even in cases where the dynamics of large quantities of molecules synchronize with the external signal. On the contrary, during the time period P, molecules have the chance to form compounds or dissociate multiple times, and these events, induced by the faster molecular dynamics, account for most of the statistics shown in the WTDs.

The increase of the magnitude M of the perturbation resulted in an increase of the number of molecules activated which can be clearly observed from the variations in the non-normalized histograms, but this does not always correspond to a significant change in the distributions of the SIP.

Increasing the period P implies larger lapse of time in which the mechanotransduction is switched on. Consequently, a larger number of events are expected. If the dynamics of the system does not change during the oscillations of the perturbation, then the area under the histograms increases with the increase of P, while the WTDs do not change.

#### Ageing

The integral distribution and the WTD are quantities computed over a time period are named the epoch, and are much larger than the support of the represented distributions but smaller than the total length of the simulation. Because the system is in a non-equilibrium condition, we cannot state that the WTD does not depend on the epoch so the WTD can be rewritten as *ψ*_*t*_(*τ*) where *t* is the initial time of the epoch of fixed extension used for the computation. Larger epochs reduce the lack of statistics and noise, while smaller epochs allow us to compute *ψ*_*t*_(*τ*) by choosing the age *t* from a larger range of available values. We tested different epoch sizes, and we compromised for a period of 10,000 s.

Ageing is characteristic of all the interacting agents, even though it is not so easy to estimate the effects of the passage of time in the histograms and WTDs, due to large confidence intervals and noise therein. Indeed, for some parameters and molecules, both the histograms and WTDs show alternate upsurge and decline patterns, and irregular cyclical variations. Even if the system is maintained in an out of equilibrium condition, the dynamics of the system responds and adapts accordingly to a mechanical perturbation.

Ageing is a direct consequence of the initial conditions; nevertheless, given that the system is regularly far from the equilibrium, ageing is prevalently due to a variation of regimes in the dynamics of the system. Therefore, the state of the system at t = 0 should not be seen as a static initial condition, but as if the system reached such a state due to a specific long standing dynamics. Indeed, we have simulated a case where the cell had been stimulated with two consecutive trains of oscillating perturbations with different frequencies. The resulting histograms show there was no ageing at the end of the first perturbation and that ageing appeared during the second regime.

## Discussion

The study illustrated that within the noise of mechanotransduction lies important information relating to the dynamics of the process. This was observed with alteration of mechanical stimuli, and modification of ERK pathway feedback loops. The study also identified innovative molecular targets, thus new therapeutic approaches to develop for osteoporosis. These involve the mimicry of mechanical stimulation, at the mechanotransduction level, by interfering with the interactions and activation states of the Raf-MEK-ERK signalling module. This was feasible using an analysis via WTD method which detected eloquent information from the noise of mechanotransduction.

### Implication of simultaneous modulation of mechanical load and mechanotransduction

The dynamics of various molecules involved in an OB’s mechanotransduction has been addressed in the Mech-ABM. Whereby the mechanical stimulation was modulated in terms of magnitude and period of activation (P), i.e. load frequency; while with respect to mechanotransduction, adjustments were by altering activation-deactivation cycles (i.e. feedback loops). Preliminary analyses on the resulting signals showed the external mechanical perturbation functions as a switch, but did not suggest any further synchronization on time scales smaller than the time period of the external mechanical loads. The magnitudes of activated molecules increased with respect to the value of mechanical load applied at the tissue level, specifically proteins such as phosphorylated ERK (pERK), in line with physiological observations [54, 55]. The magnitudes of molecules in time were driven by a slower dynamics frequently interrupted by asynchronous large fluctuations. The reported observations particularly with respect to the magnitude and oscillatory trend of pERK, are similar to what Grabowski et al. demonstrated with respect to pERK pulsating activity [56]. Their work showed that pERK activation pattern is linked to the stimuli’s mode, though in their study the stimuli was epidermal growth factor (EGF) not mechanical. Nonetheless, Grabowski examination of the influence of different feedback loops illustrated that SIP is a mechanism by which cells can transmit information at high bit rates. This mechanism is equivalent to neuronal information processing. Considering that previously OB and OCy interpretation of mechanical signal was linked to neuronal response to excitatory events [57–59], the significant role SIP play in OB’s mechanotransduction becomes clear, i.e. the utilisation of SIP to code for, and store previous mechanical information. It is worthy of note that though mechanical load altered the magnitude and modality of activated molecules, it did not significantly alter the modalities in terms of WTDs. Changes in the WTDs were significant due to the small local error estimated from the distributions. Conversely, the frequency of applied load had more significant impact on shifting mechanotransduction dynamics, in terms of WTDs and their modalities (seen more evidently in Fig. 5). This emergent behaviour is also observed under physiological conditions where load frequency has more impact on bone formation [60–62].

The WTD of Raf, MEK and ERK molecules presented multimodality which highlights the presence of preferred SIP in the occurrence of events. Changes in the activation and deactivation cycles between molecules in the RAF-MRK-ERK module showed variations in WTDs of molecules belonging both to the classes near one of the perturbed edges and to other modules of the mechanotransduction network. For example, when *P* = 1000 s, the WTD of RAF bound to MEK was characterized by a bimodal distribution for low values of active MEK dissociation times, while it became unimodal at larger value of the same parameter. This is of significance due to the role of ERK module in mechanotransduction and the emergence of molecular memory [63, 64]. Furthermore, this is in line with Mitra et al. predictions that the oscillatory behaviour and SIP within the MAPK pathway form a mechanism where the cell stores previous stimuli as “short memory” [65]. Their observations were attributed to the flux imbalance of proteins Raf-MEK-ERK in their phosphorylation cycles, which is what Mech-ABM simulated by modulation of mechanotransduction activation cycles. Moreover, the concept of mechanical molecular memory has been described and demonstrated by Yang et al., however, their initial work only demonstrated that at the level of the transcription factor YAP/TAZ. Nonetheless the cascade linking mechanoreceptors, such as integrins, and YAP/TAZ has not yet been fully characterised, though ERK activation was previously linked with modulation of ECM stiffness. Our previous work with Mech-ABM further emphasises the link between mechanoreceptors and emergence of molecular mechanical memory, through the activation dynamics of pERK [32]. This is promising considering that recently Yang JM et al. demonstrated that ERK and its cascade are pivotal in linking mechanical and chemical signal via ERK oscillatory activation dynamics [55]. Our study forecasts that Raf-MEK-ERK module is important for mechanical-information transfer, decoding it as SIP, and providing a signature for mode of mechanotransduction.

Furthermore, via the Mech-ABM and the analysis method, shifts in WTDs’ modalities were observed at the level of gene expression events. These were reflected by many of the mRNAs WTDs which were affected by ageing, a variation of the shape from a bimodal to trimodal distribution, showing the emergence of a new preferred SIP of events manifestation. Variation in the modes implies variations in the dispersion times between chained events of the molecular network.

Currently molecules such as PTH analogue (iPTH) are used as a treatment for osteoporosis to trigger an increase in bone mass. The current view, based on the principle of mechano-regulation proposes that combining mechanical and biochemical treatment (via iPTH) can achieve optimal bone formation. The PTH receptor (PTHR) in OBs was demonstrated to recruit MEK-ERK during its endocytosis, impacting OB activation overtime via PTHR recruitment of β-arrestin scaffold proteins [66, 67]. In the presented study, the Raf-MEK-ERK module was shown to act as gatekeepers for mechanotransduction when mechanical load is applied, which is a growing view in bone mechanobiology [57, 63, 64, 68–72]. The study also forecasted that modifying the module activation can significantly shift mechanotransduction and overall state of the system. Hence we propose that the design of small molecules interfering with the Raf-MEK-ERK module activation, by perturbing their binding-interaction will allow for a shift in mechanotransduction, which can significantly amplify OB response to mechanical stimulation. These could be achieved by interfering with their interaction with scaffold proteins, such as β-arrestins, to target PTHR signalling; or Paxillin and GIT1 to affect focal adhesions signalling. Consequently, osteoporotic patients will require very mild mechanical activation, if any, via localised vibrating devices to induce a therapeutic effect in combination with anabolic treatment such as iPTH. This is plausible considering the advances in developing small-molecule drugs interfering with intracellular scaffold proteins, and their success in clinical trials [73–75].

### Analysis of mechanotransduction noise

In this presented work, the abrupt fluctuations departing from the trend have been explored, analysed and presented as modalities in terms of magnitude of active molecule and WTDs. This has allowed for extraction of hidden information characteristic to each class of molecule, and overall mechanotransduction dynamics. Activation of intracellular mediators is reliant on availability of molecules and their activation states [22]. The underlying mechanisms resemble a chained Feynmans blending single molecules into signals and fluctuations at the meso/macroscopical scales. Thus finding the absolute maxima within multiple activation phases or mechanical triggers is not trivial. Consequently, a time average operator was applied as a filter to define significant mechanotransduction events, which was more appropriate in comparison to a standard ensemble average [76]. Therefore, there are no real means for the molecules with a finite interaction range to perceive a global average signal. Hence, for a biological complex system, a time average operator is more meaningful for comparing fluctuations against the trend of the system. As the system demonstrated an average molecular signal that is intermittent in time it exhibited non-ergodicity. Thus, this necessitated transformation of the filtered signal, where the trend around the signal was removed to acquire fluctuations around the trend. In many physical and biological systems the variation of a signal is delayed by the presence of internal structures and processes that are not easily accessible to experimental observations [47]. Therefore, in these cases, the approach in the study dealt with signals showing extra layers of complexity. Therefore, the disentangling of the subordinated process is not unique, and so the preferred approach was to address the problem numerically by the identification of critical events in the system under analysis. It is reasonable to define critical events as the large and abrupt fluctuations in each molecular species due to the stochasticity of the events that the Mech-ABM captures. This is in contrast to more traditional approaches, where noise has been adopted as a measure to quantify the error around the expected values of time dependent signals [76, 77].

### Limitations

The model had generated plausible predictions regarding the shift in the dynamic of cell response to mechanical stimulation when the mechanical stimulus and intracellular feedback loops were altered. We acknowledge that some assumptions made within the model will impose a level of constraint on how the data is interpreted, for instance that an OB is a spherical cell and that all intracellular molecules involved are homogeneously distributed within the cytoplasm and the nucleus. Another noteworthy limitation is that our data is illustrating the dynamics of a single OB, while bone formation at a spatial location within bone is driven by a collection of OBs. Additionally, what control BR process is the cell-cell interaction between OBs, OCys and OC, which is outside the scope of the Mech-ABM. It will be interesting to see if upscaling the presented findings to a population of many OBs interacting with OCys and OCs will induce a nudge effect that will ultimately enhance bone formation in different sites in bone tissue.

We also acknowledge that the model did not include all cell signalling mechanisms which drive bone formation, particularly WNT signalling. However, given that WNT and ERK pathway crosstalk, therefore our integration of a black-box approach to simulate ERK feedback loops, introduces WNT influence on ERK to some degree. Nonetheless, the effect of ERK signalling on WNT could not be integrated as it is not part of the mechanotransduction pathway. Yet, the Mech-ABM can be extended to include these elements easily for future investigations to study the impact of WNT on mechanotransduction and ultimately bone formation.

## Conclusions

This study simulated modulation of mechanical load and alterations in mechanotransduction feedback loops, on cellular response to mechanical stimulation. The study demonstrated that WTD of some molecule, particularly within the ERK cascade, is a dynamic marker which can be used as a signature for the system’s dynamics in normal and pathological conditions. Consequently hidden information characteristic to each class of molecule, particularly of the ERK pathway, and of the process dynamics were extracted. The presented method of analysis is a suitable tool to identify discrete variations in mechanotransduction dynamics attributed to the pathophysiology of osteoporosis. Additionally, the analyses can be used in the future to complement in vitro experiments tailored to explore spatiotemporal effects of treatments on mechanotransduction, and if they mimic bio- mechanical stimuli.

## Supplementary information

**Additional file 1: Figure S1.** Cytoplasmic and nuclear biochemical reactions.

## Data Availability

We will release our code, data and appropriate materials under the Creative Commons Attribution License. These are available on Google Drive at this link: https://drive.google.com/drive/folders/1Q0HFuvmv6NTxpBXfKQerR93I-4_t9LAu?usp=sharing

## Abbreviations

ABM: Agent-based model
ACS: Activation Cycle Switch
BR: Bone remodelling
ECM: Extracellular matrix
EGF: Epidermal growth factor
ERK: Extracellular signal-regulated kinase
iPTH: PTH analogue
Mech-ABM: Hybrid mechanical-ABM model
MEK: MAPK/Erk kinase
OB: Osteoblast
OC: osteoclast
OCy: Osteocyte
pERK: Phosphorylated ERK
PTHR: PTH receptor
Raf: Rapidly Accelerated Fibrosarcoma
SIP: Signal interim period
WTD: Waiting Time Distribution
