# Supplementary material for: Revealing hidden information in osteoblast’s mechanotransduction through analysis of time patterns of critical events
Source: BMC Bioinformatics. 2020 Mar 18;21:114. doi: 10.1186/s12859-020-3394-0 (PMC7079370; doi:10.1186/s12859-020-3394-0)
Supplement: Supplementary file 1 — Additional file 1: Figure S1. Cytoplasmic and nuclear biochemical reactions. [file 12859_2020_3394_MOESM1_ESM.docx]

# Supplementary material

FLAME

FLAME is capable of generating C based parallelisable code executable on different parallel hardware architectures, was used to create and simulate the model. FLAME implements a discrete time-fixed sweep update scheme meaning in the simulation, that the time advances at each iteration of a fixed amount defined as time unit in which all agents are updated only once in an internal defined order

## Supplementary figure 1


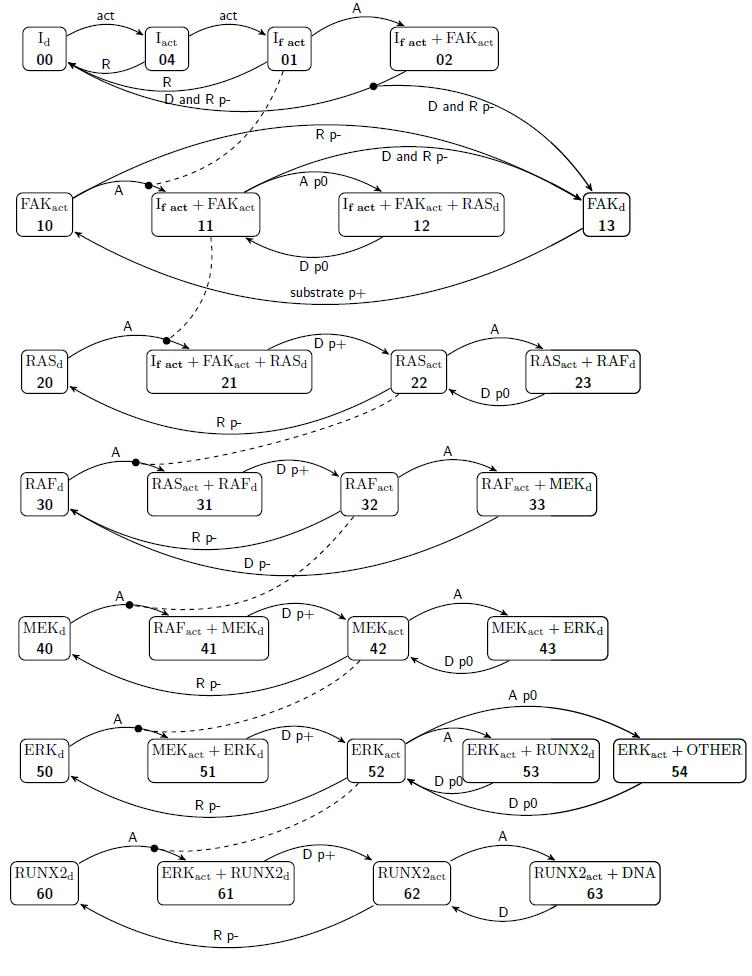
 **Cytoplasmic and nuclear biochemical reactions.**  A=association, D=dissociation, R= reactivation cycle, p+=phosphorylation, p-=dephosphorylation, p0=no phosphorylation, act= active, d = dormant, rand=chosen at random
